# Lattice Strain Evolutions in Ni-W Alloys during a Tensile Test Combined with Synchrotron X-ray Diffraction

**DOI:** 10.3390/ma13184027

**Published:** 2020-09-11

**Authors:** Tarik Sadat, Damien Faurie, Dominique Thiaudière, Cristian Mocuta, David Tingaud, Guy Dirras

**Affiliations:** 1 LSPM-CNRS UPR3407, 99 Avenue Jean-Baptiste Clément, Université Sorbonne Paris Nord, 93430 Villetaneuse, France; faurie@univ-paris13.fr (D.F.); david.tingaud@univ-paris13.fr (D.T.); dirras@lspm.cnrs.fr (G.D.); 2 Laboratoire d’Automatique, de Mécanique et d’Informatique Industrielles et Humaines (LAMIH), UMR CNRS 8201, Université Polytechnique Hauts-de-France, F-59313 Valenciennes, France; 3 Synchrotron SOLEIL, L’orme des Merisiers, Saint Aubin BP 48, 91192 Gif-Sur-Yvette, France; dominique.thiaudiere@synchrotron-soleil.fr (D.T.); cristian.mocuta@synchrotron-soleil.fr (C.M.)

**Keywords:** metallic composites, synchrotron X-ray diffraction, Ni, Ni-W alloys

## Abstract

Ni and Ni(W) solid solution of bulk Ni and Ni-W alloys (Ni-10W, Ni-30W, and Ni-50W) (wt%) were mechanically compared through the evolution of their {111} X-ray diffraction peaks during in situ tensile tests on the DiffAbs beamline at the Synchrotron SOLEIL. A significant difference in terms of strain heterogeneities and lattice strain evolution occurred as the plastic activity increased. Such differences are attributed to the number of brittle W clusters and the hardening due to the solid solution compared to the single-phase bulk Ni sample.

## 1. Introduction

Because of their good mechanical properties such as high hardness and wear resistance [1], nickel-tungsten (Ni-W) alloys are competitive materials that can be used to replace chrome deposits. In addition, they have better magnetic [2], tribological [3], corrosion [4,5,6], and electrical [7] properties. These alloys are generally produced by electrodeposition (ED) [8,9,10,11,12,13,14,15,16,17], magnetron co-sputtering [18], mechanical alloying [19], sintering processes [1,7], and thermal plasma-processes [20]. Due to diffusion of the body-centered cubic (bcc) tungsten phase (W) in the face-centered cubic (fcc) nickel one (Ni), a Ni(W) solid solution is commonly obtained by such processes [7,8,9,10,11,12,13,14,15,16,17,18,19,20,21]. The dissolution of tungsten atoms in the nickel lattice causes a shift of the fcc Bragg peaks towards lower scattering angles [7,13,19,22,23]. Based on Vegard’s law [24], there is a linear relationship between the lattice parameter of the Ni(W) fcc phase and the W content in the solid solution [1,7,13,19,25]. Recently, the production of a bulk (Ni + W) composite-like microstructure by blending controlled amounts of high-purity Ni and W powder particles was successfully achieved by spark plasma sintering (SPS) [1,7,26,27]. Synchrotron X-ray diffraction using a high brilliance source is widely employed to analyze bulk metal samples. The mapping of the distribution of the precipitate microstructure [28], the investigation of the dynamic interactions during solidification of alloys [29], and the characterization of the evolution of phases during aging treatments [30] are some well-known applications. To determinate the influence of the Ni(W) solid solution versus the W phase on the macroscopic deformation of Ni-W alloys and in order to investigate the strain distributions in a Ni-30W (wt%) alloy sintered by SPS, in situ X-ray diffraction (XRD) experiments were performed in [27]. Five separate domains were identified to describe the whole mechanical behavior better. It has been clearly shown that during uniaxial tensile deformation, the cracks propagated inside W aggregates and were stopped at the Ni(W)/W interface at a macroscopic strain of 5% (corresponding to about 620 MPa) [27]. To better understand the intragranular heterogeneities between the two phases, we compare in this continued work, crystal lattice strains in Ni(W) and Ni phases, for different W amounts (Ni-10W, Ni-30W, and Ni-50W (wt%)). The Ni(W) {111} solid solution strain evolution during a uniaxial tensile test is studied and compared to that of the pure Ni {111} phase by using the same in situ tensile tests combined with XRD. In addition, we analyzed the plastic and fracture behavior using in situ monitoring of the full width at half maximum (FWHM), which has not been done previously for these systems to the best of our knowledge.

## 2. Materials and Methods

Bulk samples were sintered by the SPS technique. This is a fast-process sintering and high-temperature technique that provides a fast heating and cooling rate with a short consolidation time and controllable pressure [28]. A uniaxial load is applied to graphite dye that contains the powder. It is heated by the Joule effect via a pulsed current [29]. The details on the SPS parameters are published elsewhere [7].

The in situ experiments were carried out at the French radiation synchrotron facility (SOLEIL) on the Diffabs beamline with a six-circle diffractometer. A Deben^TM^ tensile stage was used to perform the tensile tests. Tensile tests were conducted using a step-by-step loading procedure at room temperature at a strain rate of 2 × 10^−3^ s^−1^. The Digital Image Correlation (DIC) technique was used to obtain an accurate value of the sample macroscopic strain. Aramis software [30] was used to analyze the images. The monochromatic beam’s energy was fixed at 18 keV, corresponding to a wavelength of about 0.68 Å. The size of the beam was set to 300 × 242 µm^2^, and the incidence angle was fixed at 10°. A scintillator was used to adjust the sample height with respect to the beam at each deformation step (the vertical position is tailored by keeping half of the direct beam intensity). An XPAD-S140 two-dimensional detector [31] was mounted at about 64 cm from the sample surface to acquire the 560 × 240 px^2^ 2D diffractograms. A picture of the whole set-up can be found in a previous paper [27]. For each experimental data point, the corresponding lattice strain ε is calculated using the unloaded state as the reference one:

$$\varepsilon =\ln\;\left(\frac{d_{hkl}}{d_{0}}\right)=\ln\;\left(\frac{\sin\left(\theta_{0}\right)}{\sin\left(\theta_{hkl}\right)}\right)$$

where $d_{hkl}$ and $d_{0}$ are the interplanar distances of the respective loaded and unloaded states and $\theta_{hkl}$ and $\theta_{0}$ the angular positions of the considered diffraction peak (*hkl*) in the respective loaded and unloaded states. The strain perpendicular to the tensile direction was analyzed. In this study, the peaks shifted towards the higher Bragg angle position during the tensile test, which means that the interplanar distance $d_{hkl}$ decreased due to the macroscopic deformation.

## 3. Results and Discussion

**Figure 1:**
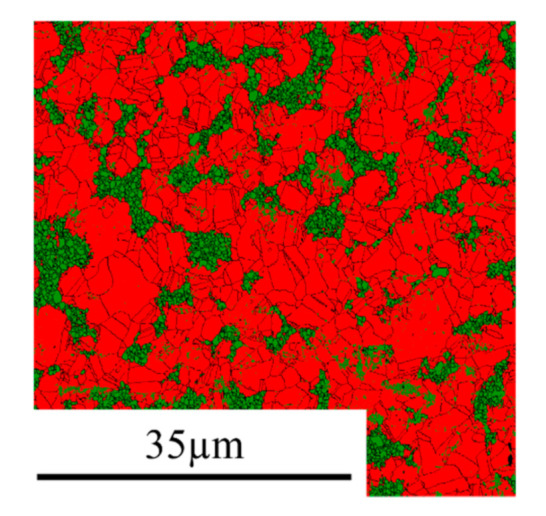
EBSD phase map of the Ni-50W alloy; the Ni(W) solid solution and W clusters appear in red and in green, respectively. (For interpretation of the references to color in this figure legend, the reader is referred to the web version of the article [7]).

As already discussed in [7], the Ni-30W and Ni-50W alloys are made of fine-grained multi-crystalline clusters of W (average grain size of about 0.5~0.8 µm) surrounded by randomly oriented grains of Ni(W) (average grain size between 3.9 µm and 8.4 µm depending on the initial amount of W). The Ni-10W was found to be composed of the sole Ni(W) phase (no W cluster). It was established that the average grain size of the Ni(W) phase within the alloy decreased significantly with an increasing amount of W. The bulk Ni exhibited coarse grains with an average grain size of 19.2 µm [7]. To illustrate the microstructure of the alloys, Ni-50W Electron backscatter diffraction (EBSD) phase map is presented in Figure 1 where the Ni(W) solid solution and W clusters appear in red and in green, respectively (other phase maps can be found in [7]).

**Figure 2:**
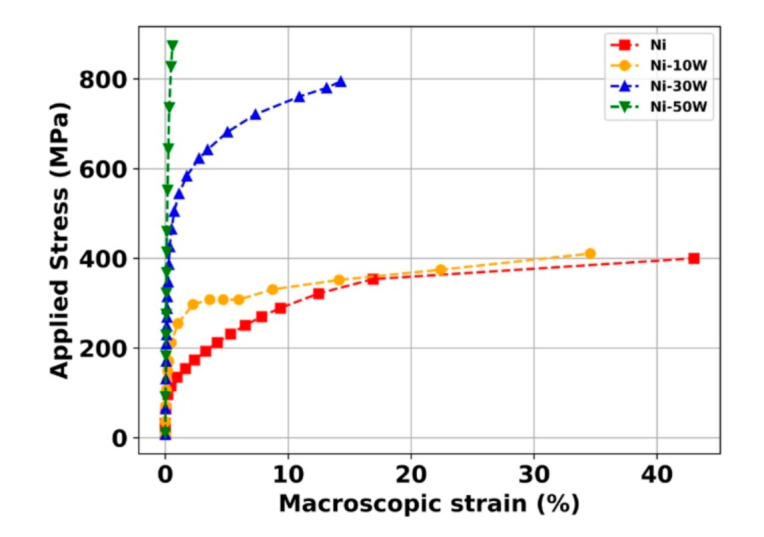
Applied stress as a function of the macroscopic strain of the bulk Ni and Ni-W alloys.

Applied stresses as a function of applied strains (measured by DIC) are presented in Figure 2. As seen, the Ni-30W alloy displays the best combination of both ultimate tensile strength (UTS) (800 MPa) and uniform strain. As expected, the bulk Ni is the sample that deformed the most but was also the one that highlighted the lowest value of UTS (400 MPa). On the contrary, the Ni-50W exhibited the highest UTS (919 MPa) at the expense of ductility. Indeed, it is well known that the addition of W leads to an increase in UTS at the expense of the uniform elongation.

**Figure 3:**
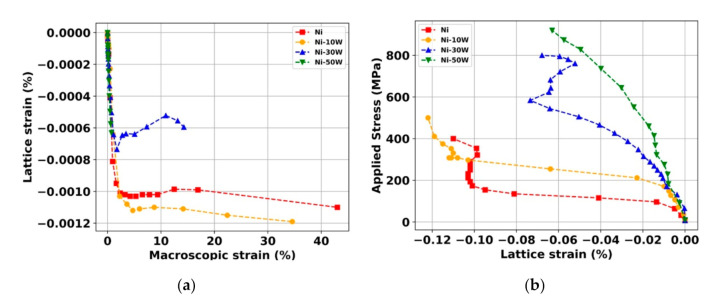
(**a**) Ni {111} or Ni(W) {111} lattice strain as a function of the macroscopic strain of bulk Ni and Ni-W alloys. (**b**) Applied stress as a function of the Ni {111} or Ni(W) {111} lattice strain of the bulk Ni and Ni-W alloys.

Figure 3a illustrates the lattice strain evolution of the Ni {111} and Ni(W) {111} of the bulk Ni and Ni-W alloys as a function of the applied strain computed by the DIC technique. Figure 3b displays the applied stresses as a function of the lattice strains of Ni {111} or Ni(W) {111} of the bulk Ni and different Ni-W alloys. It is interesting to notice that the elastic domain is well established for all the tracked lattice strains. Indeed, the linearity of the curves occurs at the beginning of the deformation and lasts more or less as the applied stresses increase. It is worth noting that the lattice strains are negative due to the fact that only the crystallographic orientations perpendicular to the tensile tests are probed in this work. Regarding the Ni-10W alloy and the bulk Ni, the curves are characterized by three or four distinct domains:

(i) From 0 MPa to 75 MPa (for the Ni) and 180 MPa (for the Ni-10W), the lattice strains increase linearly with the applied stresses. This is in accordance with the elastic domain at the macroscopic scale highlighted in Figure 2.
(ii) At some points, from 75 MPa to 170 MPa (for the bulk Ni) and from 180 MPa to 290 MPa (for the Ni-10W alloy), a sudden change in lattice strain occurs.
(iii) From 170 MPa to 350 MPa (for the bulk Ni) and from 290 to 350 MPa (for the Ni-10W alloy), a very slight variation of the crystal lattice occurs.
(iv) The last points are associated with a sharper increase, in absolute value, of the crystal lattices, most probably due to the sample’s striction.

Domain (ii) is quite surprising. We did not find in the literature such variations of about 0.1% for polycrystalline materials with micrometric grain size distribution [31,32]. The lattice strain is generally smoother beyond the elastic domain (of the order of 0.1%). This effect might be attributed to the internal microstructure of our materials designed by SPS. Indeed, the disorientation of the grain boundaries of the Ni and Ni(W) phases has been characterized by electron back scatter diffraction (EBSD) analysis [7]. A relatively significant fraction number of Σ = 3 boundaries (Σ3) was observed, showing an ideal misorientation angle of 60° around the <111> axis and including coherent {111} and incoherent {112} twin boundaries. For example, the fraction number of Σ*3* boundaries was equal to 40.5% and 52.4% for the bulk Ni and the Ni-10W alloy, respectively. It has been reported, in the recent literature, that after the mechanical deformation, at the post mortem state, a significant decrease of the initial fraction of Σ*3* boundaries occurs. Such a phenomenon is due to an intense dislocation activity and interaction with those Σ*3* boundaries [33]. This phenomenon seems to be more severe in the case of pure Ni as compared to the Ni(W) phase. It is remarkable that such evolutions are imperceptible at the macroscopic scale presented in Figure 2.

Regarding the Ni-30W alloy, which has been studied in previous work [27], five separate regions are identified in the evolution of the lattice strain. Furthermore, as it has been demonstrated in [27], the cracks propagating inside W aggregates are stopped at the Ni(W)/W interface for a macroscopic strain equal to 5% (corresponding to about 620 MPa). We now turn to the Ni-50W alloy, which presents a different lattice strain evolution of the Ni(W) {111} orientation. Here, we can only distinguish two domains: 

(i) An elastic domain up to 400 MPa of the applied macroscopic stress;

(ii) An elastoplastic domain up to 900 MPa of the applied macroscopic stress and, finally, the sample’s failure.

**Figure 4:**
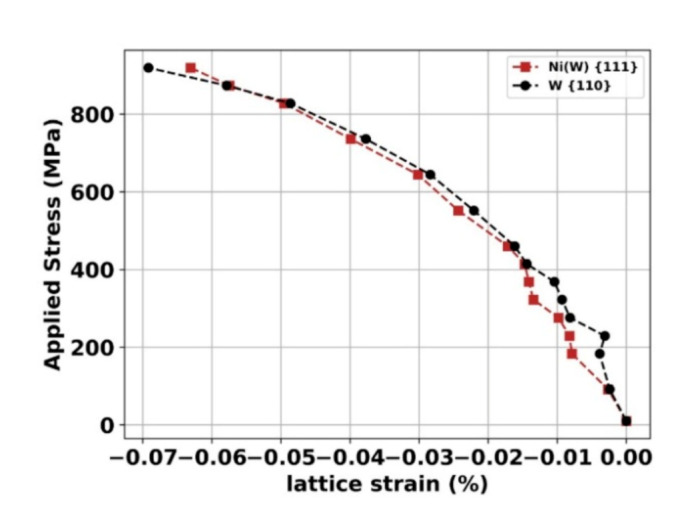
Applied stress as a function of the lattice strain of the Ni(W) {111} and W {110} orientations of the Ni-50W alloy.

The applied stress as a function of the lattice strain of the Ni(W) {111} and the W {110} orientations of the Ni-50W alloy is presented in Figure 4. The brittle fracture is well established in both cases. Around 870 MPa, an inversion of the curve occurs; a load-transfer seems then to take place close to the failure of the sample from Ni(W) to the W phase.

**Figure 5:**
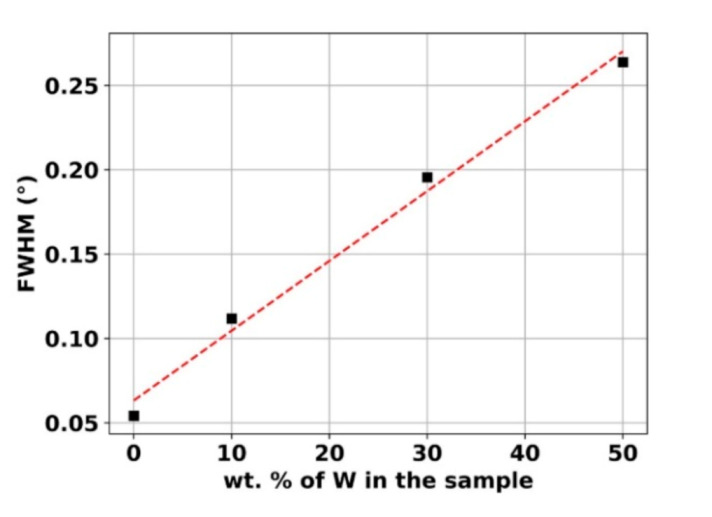
Evolution of the full width at half maximum (FWHM) of the Ni {111} or Ni(W) {111} as function of the weight percentage of W in the sample of the bulk Ni and Ni-W alloys in the unloaded state.

To gain more insight into the influence of the W amount, we present in Figure 5 the evolution of the FWHM of the Ni or Ni(W) {111} orientation in the unloaded state as a function of this amount (from 0 to 50 wt.%). A linear increase of the FWHM is clearly observed. Such an evolution is in accordance with the grain size decreasing of the Ni and Ni(W) phases with the amount of W [7].

**Figure 6:**
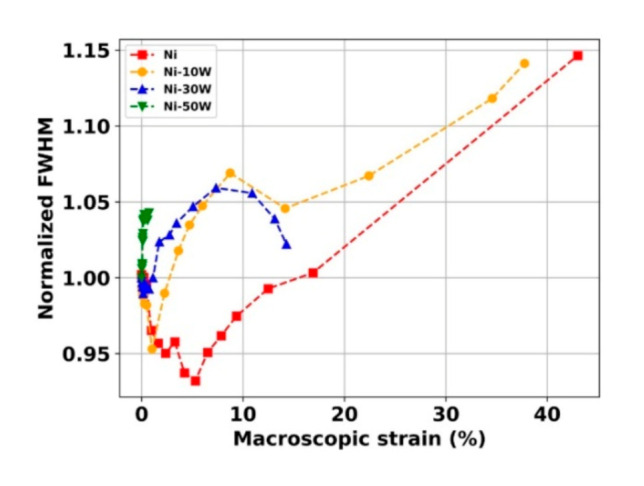
Normalized FWHM of the Ni {111} and Ni(W) {111} as a function of the macroscopic strain of the bulk Ni and Ni-W alloys.

Finally, the evolutions of the FWHM with the macroscopic strain were analyzed. Normalization with the unloaded state value was first performed, and the as-obtained curves are presented in Figure 6.

As can be seen, the FWHM evolves in the same way for Ni and the Ni-10W alloy. Indeed, in the elastic domain and at the beginning of the plastic domain activity, a decrease is observed (up to 5% for the Ni and 2% for the Ni-10W) which can be attributed to decreased strain heterogeneities inside those materials. Such a phenomenon might be attributed to a relaxation of internal stresses and induced by the tensile test. Another explanation might be the decreasing of the Σ*3* boundary fraction leading to an increase of the coherent diffraction domains and finally, an increase of the FHWMs. After that, the FWHMs reach a minimum and then seem to follow an increasing tendency with the applied strain up to failure. This increase of the FWHM is synonymous with an increase in deformation heterogeneities due to the dislocation activity, which increases with the plastic deformation, which is classical in the case of coarse-grained materials [34].

The Ni-30W alloy has a more singular behavior. At the beginning of the test, the FWHM decreases up to 1% of applied strain, which might also be related to the decreasing of the Σ3 boundary fraction in the Ni(W) phase during the tensile test. Such a decrease was quantified from post mortem EBSD analysis in [7]. After that, the FHWM increases because of dislocation activity during plastic deformation, and then, from 7% applied strain, it decreases until failure. Indeed, the crack propagation from the brittle W aggregates to the Ni(W) matrix induces a mean stress relaxation in the Ni(W) phase that explains this decrease [27].

The FWHM evolution of the Ni-50W alloy is much different. An increase at the beginning of the macroscopic deformation (contrarily to the other ones) is first observed. It is then followed by a plateau, difficult to distinguish at this scale. In this sample, with the highest amount of the bcc phase, elastic interactions might be stronger between the phases than in the Ni-30W alloy. Moreover, the cc phase’s stresses are more pronounced due to a higher theoretical Young’s modulus (400 GPa), which can explain such dissimilarity. Regardless, this sample is globally fragile because the W-phase is continuously distributed in the material, which allows the cracks to propagate quickly throughout the material [7].

## 4. Conclusions

Ni and Ni-xW alloys (x varying between 10, 30, and 50 wt.%) were mechanically deformed in situ under synchrotron XRD. Differences in terms of lattice strains of the Ni {111} or Ni(W) {111} crystallographic orientations were identified. A significant increase in lattice strains between 75–170 MPa (for the bulk Ni) and 180–290 MPa (for the Ni-10W) occurred. Such a phenomenon might be correlated to a decrease of the Σ*3* boundary fraction during the tensile tests. A load transfer between the Ni(W) phase and the W one was observed in both Ni-30W and Ni-50W alloys. The full width at half maximum (FWHM) of the samples decreased (up to 5% mechanical deformation for the Ni and 2% for the Ni-10W one), which can be attributed to a decrease of strain heterogeneities inside those materials. These in situ observations based on X-ray diffraction are relevant to target in situ transmission electron microscopy (TEM) analysis to observe the dislocations and interaction with the Σ*3* boundaries and the crack propagation.
